# Genetic determinants of the molecular portraits of epithelial cancers

**DOI:** 10.1038/s41467-019-13588-2

**Published:** 2019-12-11

**Authors:** Youli Xia, Cheng Fan, Katherine A. Hoadley, Joel S. Parker, Charles M. Perou

**Affiliations:** 1 Curriculum in Bioinformatics and Computational Biology, University of North Carolina at Chapel Hill, Chapel Hill, NC USA; 2 Department of Genetics, University of North Carolina at Chapel Hill, Chapel Hill, NC USA; 3 Lineberger Comprehensive Cancer Center, University of North Carolina at Chapel Hill, Chapel Hill, NC USA; 4 Department of Pathology and Laboratory Medicine, University of North Carolina at Chapel Hill, Chapel Hill, NC USA

**Keywords:** Cancer genomics, Cancer genomics

## Abstract

The ability to characterize and predict tumor phenotypes is crucial to precision medicine. In this study, we present an integrative computational approach using a genome-wide association analysis and an Elastic Net prediction method to analyze the relationship between DNA copy number alterations and an archive of gene expression signatures. Across breast cancers, we are able to quantitatively predict many gene signatures levels within individual tumors with high accuracy based upon DNA copy number features alone, including proliferation status and Estrogen-signaling pathway activity. We can also predict many other key phenotypes, including intrinsic molecular subtypes, estrogen receptor status, and *TP53* mutation. This approach is also applied to TCGA Pan-Cancer, which identify repeatedly predictable signatures across tumor types including immune features in lung squamous and basal-like breast cancers. These Elastic Net DNA predictors could also be called from DNA-based gene panels, thus facilitating their use as biomarkers to guide therapeutic decision making.

## Abstract (web summary)

Effective precision medicine strategies rely on the ability to predict tumour behaviour based on molecular characteristics. Here, the authors build models to predict multiple distinct gene expression patterns using DNA copy number alterations

## Introduction

Tumorigenesis is often driven by multiple types of aberrations in DNA leading to diseases of enormous complexity and heterogeneity. The ability to dissect this heterogeneity is crucial to understanding cancer mechanisms, and for identifying patient subgroups for personalized treatments. One limitation to capture this heterogeneity lies in the characterization of disease phenotypes. With the effort of many consortiums including The Cancer Genome Atlas (TCGA), large-scale multi-platform genomic data are now available, providing an opportunity to study cancer phenotypes on a molecular level and through multiple technology types^1–4^. In particular, many gene expression signatures have been developed to define specific cancer phenotypes varying from proliferation rates to features of the tumor microenvironment^5–7^. These mRNA expression features, along with protein expression, somatic mutations, and clinical features provide a comprehensive molecular portrait of tumors. Integrating multi-platform genomic data together to elucidate the relationship between genotype and phenotype is critical to understanding genetic causes underlying tumor behaviour^8^. Building predictive models for key tumor driving phenotypes would be valuable to stratify patients for personalized treatments, especially in the clinical setting where gene expression profiling is usually not available and DNA information is routinely collected thanks to available somatic mutation gene panel tests.

In this study, we use an archive of experimentally and computationally derived gene expression signatures, alongside other well-known clinical and molecular features, as a framework to identify genetic drivers and build predictive models for solid epithelial cancer phenotypes. We use an integrative genomics approach including a genome-wide association analysis^8^, as well as an Elastic Net predictive modeling strategy^9^, to build models of complex tumor phenotypes using somatic DNA copy number alterations (CNAs). Our results identify associations between many gene expression signatures and CNAs, and between protein expression features and CNAs. Generally, we present an approach that could be applied to many other tumor types for which multi-platform genomic data are available, to evaluate the relationship between CNAs and complex phenotypes, and where predictive models of therapeutic importance could be developed using what are now common place DNA-based clinical tools.

## Results

### Characterize gene signature-specific copy number alterations

**Fig. 1:**
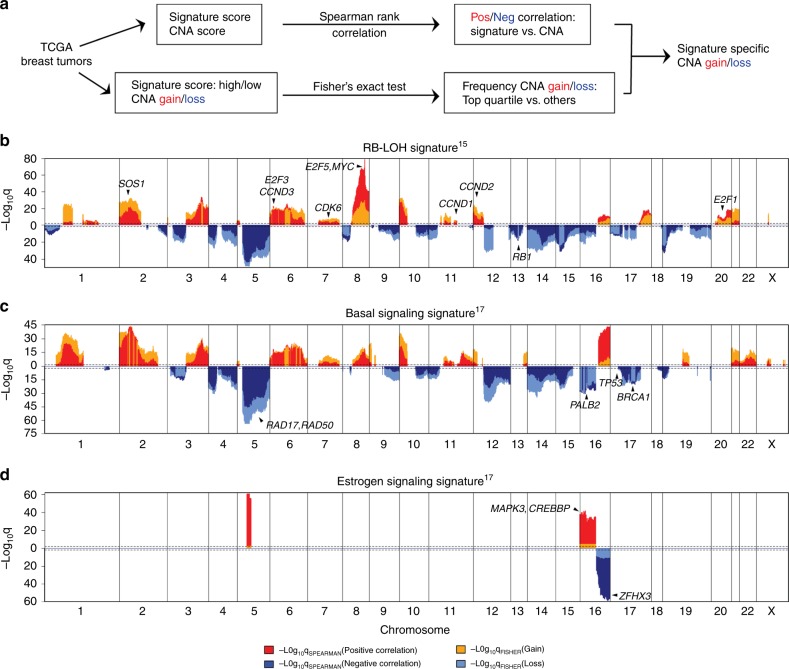
Identification of gene expression signature-specific copy number alterations in breast cancer. **a** Schematic overview of the strategy used to identify CNAs associated with gene signatures. Gain/loss indicates DNA copy number gains or losses; Pos/Neg indicates positive or negative association. **b**–**d** Spearman rank correlation was used to identify genes positively (red) or negatively (dark blue) associated with gene signatures, and Fisher’s exact test was used to compare the frequency of copy number gains (orange) or losses (light blue) for RB-LOH (**b**), Basal signaling (**c**), and Estrogen signaling (**d**) Gene Program signatures. Dashed lines indicate the significance threshold (*q* = 0.01). Only *q*-values for genes significant in both analyses were plotted. Black arrowheads indicate known pathway drivers. In each figure, chromosomal boundaries are indicated by vertical black lines.

We first aimed to investigate the possible associations between DNA copy number alterations (CNAs) and multiple gene expression signatures. We initially focused on breast tumors, where multiple gene expression signatures are already in common clinical use^10–12^. We applied a panel of 543 published gene expression signatures measuring diverse tumor phenotypes including active signaling pathways, the aforementioned known prognostic clinical models, tumor microenvironment features (i.e., immune cells, fibroblasts), and features of DNA amplicons and deletions^13^, onto 1038 breast cancers from the TCGA breast cancer project^4^ (Supplementary Fig. 1, Supplementary Data 1). DNA copy number data were used to identify possible associations linking DNA CNAs to each signature-based phenotype through a genome-wide association analysis, using a previously developed approach from a much smaller cohort of patients and a limited panel of 52 gene signatures^8^ (Fig. 1a). For each signature, we used two independent statistical methods to test for associations. We used spearman rank correlation to identify positive or negative correlations between an expression signature score and gene-level DNA segment values. We also used Fisher’s exact test to compare the frequency of CNA gains or losses in samples with high signature score (top quartile) and those with low signature score (all others). Both tests were Benjamini–Hochberg corrected to control the false discovery rate^14^. To further reduce potential false positive results, we only called a DNA CNA feature associated with an expression signature if the value was statistically significant in both analyses (*q* < 0.01). Potential DNA CNA drivers of a signature should have positive correlations and increased copy number gains in samples with high signature scores, whereas potential repressors would have negative correlations and increased frequencies of copy number losses. Through this approach, we analyzed association landscapes for each signature, noting many expression signatures had no such associations.

We looked at the reproducibility of the association landscapes by comparing our results to those from Gatza et al. for the same signatures^8^. All 52 signatures of Gatza et al. were included here, and in particular we focused on the RB-LOH signature^15^, noting that the current analysis used data on a much larger cohort of TCGA breast tumors (*n* = 1038 vs. *n* = 476); in addition, another systematic difference between the two studies is that Gatza et al. used gene expression microarrays while we used mRNA-seq. Despite these methodological differences, there was a high concordance between our association landscape for RB-LOH signature and that published by Gatza et al. (Fig. 1b); both landscapes highlighted the identification of known RB-E2F components including DNA loss of *RB1* and gains of *E2F1* and *E2F3*, as well as the amplification of multiple cell cycle drivers including *MYC* and *CCND2* (ref. ^16^).

We then examined new, and old, possible associations using all 543 gene signatures. Associations to previously determined DNA amplicon gene expression signatures were found and all encompassed regions of the corresponding amplicons (Supplementary Fig. 2), showing that the association analysis was able to identify known DNA-based drivers of expression signatures. Two important “Gene Program” universal expression signatures defined from a 12 tumor type PanCan (*n* = 3500) tumor analysis of Hoadley et al.^17^, namely a “basal signaling” signature and an “estrogen signaling” signature, both showed many informative associations. For the basal signaling signature, we identified previously known associations including loss/deletion of genes involved in DNA repair such as *RAD17*, *RAD50*, *PALB2* and *BRCA1* (Fig. 1c). For estrogen signaling signature, we identified many distinct luminal tumor DNA copy number changes including 16p gain and 16q loss^2^ (Fig. 1d). Collectively, these results demonstrate that our strategy is able to objectively find associations linking CNAs to specific gene signatures, many of which were previously known.

To further test if the associations depend on intrinsic molecular subtype, we modified the association analysis, replacing the spearman rank correlation with linear regression taking subtypes as covariates to identify universal positive or negative correlations. This led to fewer significant associations to CNAs for some signatures and the same associations for others compared to previous unadjusted results. For example, for RB-LOH signature, associations to *SOS1* and *CDK6* were no longer significant when accounting for subtype, while all associations remained significant for estrogen signaling signature (Supplementary Fig. 3). This analysis shows that molecular subtype confounds for some CNA and gene signature associations.

### CNA-based gene signature predictions by Elastic Net models

**Fig. 2:**
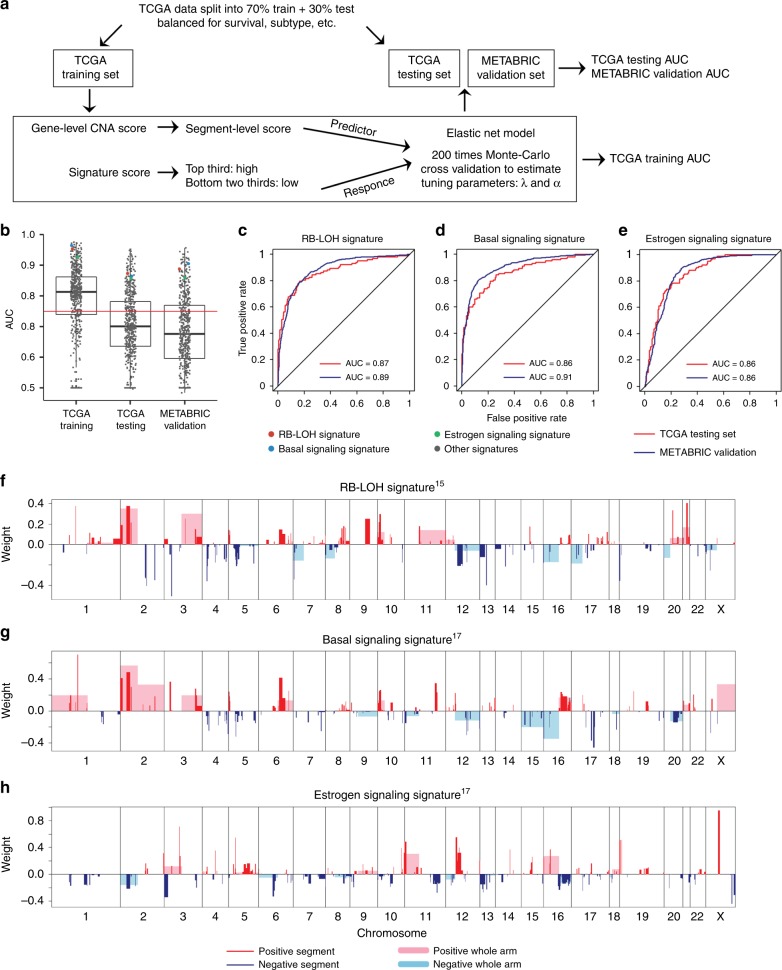
CNA-based Elastic Net prediction models for gene signatures in breast cancer. **a** Schematic overview of the strategy used to build Elastic Net models for predicting gene expression signature levels. **b** Area under curve (AUC) values for 543 signatures displayed using box and whisker plots indicating the median score (horizontal line), the interquartile range (IQR, box boundaries) and 1.5 times the IQR (whiskers); the red horizontal line indicates AUC = 0.75, which we consider to be “highly predicable”. Three signatures are highlighted with colored dots, and their feature landscapes also shown in **f**–**h**. **c**–**e** Receiving operating characteristics (ROC) curves and corresponding AUC values of TCGA test set and METABRIC validation set for predicting RB-LOH (**c**), basal signaling (**d**), and estrogen signaling (**e**) signatures. **f**–**h** Elastic net selected CNA segments and/or whole chromosomal arms and their coefficients for prediction models for RB-LOH (**f**), basal signaling (**g**) and estrogen signaling (**h**) signatures.

Given the strengths of these associations, we next sought to assess the feasibility of building computational predictors of gene expression signature levels based upon DNA CNAs features only. To successfully build predictive models, we used a statistical modeling approach called Elastic Net, which is a regularized regression model that is capable of handling large numbers of potential co-linear variables and then is able to select the most relevant features to build the final model^9^. Instead of using gene-level CNA scores as predictors, we calculated 536 segment-level CNA scores using predefined chromosome regions that have been shown to be important in cancers^18–22^ (Supplementary Data 2). These DNA segments included pan-cancer significant somatic CNAs as well as breast cancer subtype-specific CNA regions. The 1038 sample TCGA breast cancer data set was split into a balanced training set (70%) and test set (30%). Models were built solely on TCGA training set and validated on both TCGA test set as well as on a large independent breast tumor data set from the Molecular Taxonomy of Breast Cancer International Consortium (METABRIC, *n* = 1689)^3^. Models were trained to classify samples into those with high signature scores (top third) versus low signature scores (bottom two-thirds). The area under receiving operating characteristics curve (AUC) values were used to evaluate model performance (Fig. 2a).

AUC distributions for all gene signatures demonstrated high predictability for some, but not all of the signatures (Fig. 2b). 142 out of the 543 signatures had AUC values above 0.75 in both test sets, which we henceforth call as “highly predictable”. Permutation tests showed that a test set AUC of 0.75 indicates significant predictive power (Supplementary Fig. 4), noting that in permuted data the highest AUC attained was 0.63 and that the large majority were close to 0.5 as might be expected. Of these 142 signatures, only 33 were DNA-based amplicon signatures that essentially measure specific CNA events and were therefore expected to produce high AUC values. For example, signature 16q23-amplicon^23^ had the highest AUC value in METABRIC validation set (AUC = 0.96). Notably, the three signatures that we highlighted for the association landscapes, namely RB-LOH signature, basal signaling signature, and estrogen signaling signature, were all highly predictable (AUC > 0.85) as shown by corresponding receiving operating characteristics (ROC) curves (Fig. 2c–e). Among the most predictable signatures were multiple proliferation signatures and a few oncogenic pathways, whereas the least predictable signatures were mostly those representing immune infiltrates and other features of the tumor microenvironment. In particular, a HER1-C2 signature previously developed by Hoadley et al.^24^ indicating EGFR pathway activity, had AUC values of 0.90 in both test sets (Supplementary Fig. 5a). On the contrary, models for a CD8 T cell signature^25^ and a stroma signature^26^, selected no CNA features and had AUC value of 0.5.

We investigated the CNA regions selected by the Elastic Net models as a means of identifying the genetic drivers of these phenotypes (Supplementary Data 3). To directly compare versus the association landscapes, we show model feature landscapes for the three signatures. Remarkably, for RB-LOH signature and basal signaling signature, which had many associations with CNAs, there was a significant amount of overlap between the association landscape and the Elastic Net model feature landscape (Fig. 2f, g). For example, RB-E2F components as well as cell cycle components, were significantly associated with the RB-LOH signature and were selected by Elastic Net for the prediction of RB-LOH signature score. On the contrary, the estrogen signaling signature had a simple association landscape, yet the Elastic Net model selected many more features besides those in the association analysis-based regions (Fig. 2h). This suggests that Elastic Net provides additional information on the relationship between CNAs and gene signatures by taking the whole genome of data together, rather than the association analysis that evaluates genes one by one. In addition, for the highly predictable HER1-C2 signature, many EGFR pathway associated genes were selected by its Elastic Net model including *EGFR* itself, *KRAS*, *SOS1* and *NRAS* (Supplementary Fig. 5b). Taken together, our results show the ability to predict many gene expression signatures using only DNA CNAs, with high accuracy and with biological plausible and informative feature sets.

**Fig. 3:**
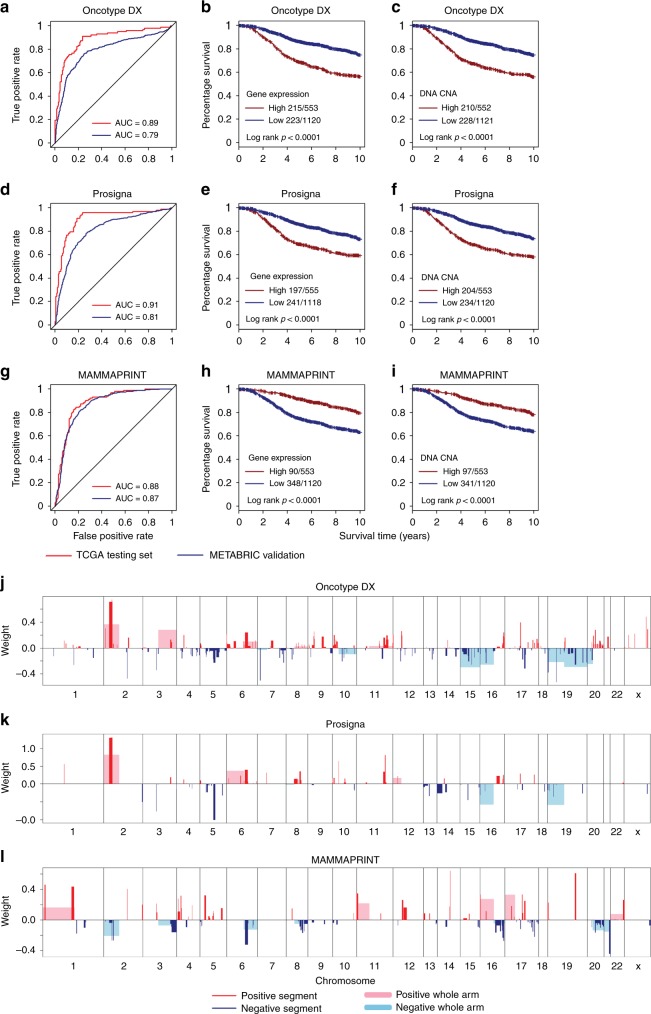
CNA-based Elastic Net prediction models for three clinically used breast cancer assays. **a**–**i** ROC curves and Kaplan–Meier curves of 10-year breast cancer-specific survival for Oncotype DX recurrence score (**a**–**c**), Prosigna risk of recurrence score (**d**–**f**) and MAMMAPRINT 70-GENE recurrence score (**g**–**i**) Kaplan–Meier curves were stratified by gene signature score (Gene Expression) and corresponding Elastic Net copy number prediction model (DNA CNA). Event statistics were indicated as number of events/total patients in both High and Low groups. **j**–**l** Elastic Net selected CNA segments and/or whole chromosomal arms and their coefficients for prediction models for Oncotype DX recurrence score (**j**), Prosigna risk of recurrence score (**k**) and MAMMAPRINT 70-GENE recurrence score (**l**).

To validate some of the key Elastic Net models with high prediction accuracy, we examined if the models correlated with patient survival in breast cancer using the large METABRIC cohort. Three research-based implementations of commercially available signatures that are commonly used in the breast cancer clinic, namely OncotypeDX recurrence score^27^, Prosigna risk of recurrence score^11^ and MAMMAPRINT 70-GENE recurrence score^28^, were all highly predictable using CNAs with corresponding METABRIC test set AUC values of 0.79, 0.81, and 0.87 (Fig. 3a, d, g); as expected, these three signatures showed strong prognostic effects as implemented by gene expression scores or DNA CNA-model based scores (Fig. 3a–i). Remarkably, models predicting OncotypeDX recurrence score and Prosigna risk of recurrence score shared many CNA regions with RB-LOH signature, indicating these two clinical assays contain features of tumor proliferation rates, which is known^11,27^ (Fig. 3j, k). Whereas the feature landscape of MAMMAPRINT 70-GENE recurrence score was similar to estrogen-signaling signature, suggesting it is more representative of estrogen signaling pathway activity (Fig. 3l). Furthermore, models for four key expression signatures that we described above (RB-LOH, basal-signaling, estrogen-signaling, and HER1-C2) all showed strong survival correlations as well (Supplementary Fig. 5c–f). These results demonstrated that successful DNA-based prediction models still retain the prognostic value of their cognate expression signatures.

### CNA-based predictions for intrinsic molecular subtypes

We next applied the Elastic Net DNA feature modeling strategy to the prediction of other complex tumor phenotypes including prediction of “intrinsic” and histological subtypes of breast cancer^11^. Prediction models for all intrinsic subtypes demonstrated high AUC values (i.e., >0.75) indicating that these RNA-based phenotypes can be well explained by DNA-based information (Supplementary Fig. 6a–d, Supplementary Data 4). The Basal-like subtype had the highest test set AUC values (>0.9), consistent with the previous findings that Basal-like breast cancers constitute a unique disease entity^17^. Regions that are frequently altered in Basal-like samples such as 1p gain and 5q loss were selected by the predictive Elastic Net model^20,21^ (Supplementary Fig. 6f). HER2-Enriched subtype also had high AUC values (>0.82), and not surprisingly, regions selected by its model included the *ERBB2* region, which is the dominant driver for this subtype (Supplementary Fig. 6g). Luminal A and Luminal B subtypes were harder to predict, yet were still highly predictable (AUC for Luminal A = 0.82, and for Luminal B = 0.76 on the METABRIC validation set). A distinct difference between these two subtypes is proliferation rate where Luminal B tumors generally have higher proliferation rate than Luminal A tumors; as might be expected, regions related to proliferation including 8q(*MYC*) amplification and *RB1* deletion were only present in the Luminal B prediction model (Supplementary Fig. 6h, i). Since the histological subtype also dictates clinical treatment decision making, we evaluated how DNA CNA-based Elastic Net model predicts two major breast cancer histology, namely invasive ductal carcinoma (IDC) and invasive lobular carcinoma (ILC) using samples that assigned to only these two histologies. ROC curves showed high AUC values in both TCGA training set (0.87) and test set (0.8) (Supplementary Fig. 6e). A hallmark of ILC, loss of *CDH1* located at chromosome 16q22.1, was reflected in the model feature landscape (Supplementary Fig. 6j).

### CNA-based predictions for individual protein expression

**Fig. 4:**
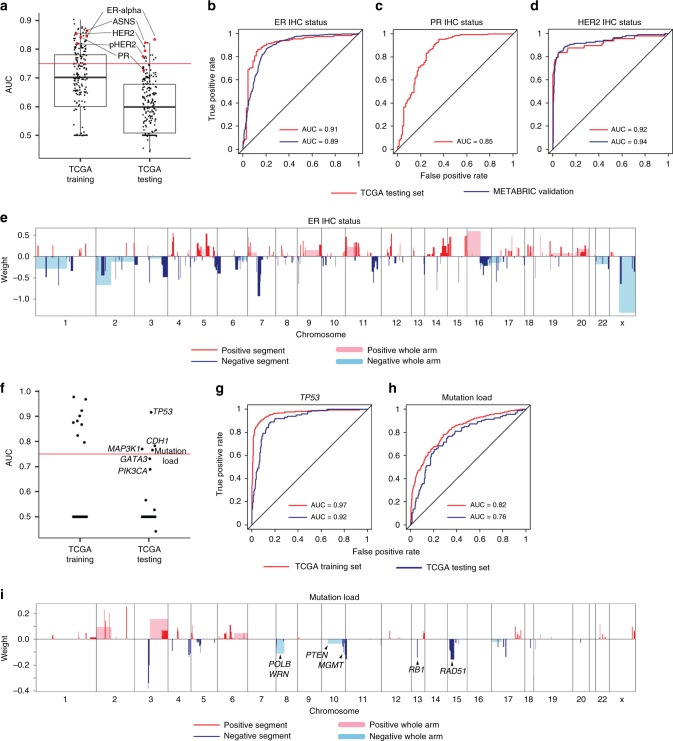
Elastic Net models predicting individual protein expression and mutation status in breast cancer. **a** Box and whisker plots indicating the median score (horizontal line), the interquartile range (IQR, box boundaries) and 1.5 times the IQR (whiskers) of AUC values for predicting protein expression of 216 proteins and phosphoproteins from TCGA RPPA arrays. Five proteins of interest are highlighted with red dots. Red horizontal line indicates AUC = 0.75. **b**–**d** ROC curves and corresponding AUC values of TCGA test set and METABRIC validation set for predicting clinical ER status (**b**), clinical PR status (**c**), and clinical HER2 status (**d**). **e** Selected CNA segments and/or whole chromosomal arms and their coefficients of prediction model for clinical ER status. **f** Dot plots indicate AUC values for predicting individual somatic mutations. Red horizontal line indicates AUC = 0.75. **g**, **h** ROC curves and corresponding AUC values of TCGA training set and TCGA test set for predicting mutations of *TP53* (**g**) and mutation load (**h**). **i** Selected CNA segments and/or whole chromosomal arms and their coefficients of prediction model for mutation load.

We further applied the Elastic Net DNA-based modeling strategy to build prediction models for individual proteins. We utilized the reverse phase protein array (RPPA) data measuring 216 proteins and phospho-proteins coming from TCGA breast cancer samples (*n* = 870)^2^. Many studies have addressed the relationship between protein levels and mRNA abundance and concluded that mRNA transcript levels predict protein levels about 50% of the time^29^. A few studies have also investigated the influence of DNA copy number on protein expression and find some proteins with significant correlations, typically those that are the target of amplification or deletion^30,31^. However, these studies assessed correlations on individual genes. Here, we used Elastic Net model building to take into account the whole genome to predict protein expression. Using the aforementioned definition of “highly predictable”, we were able to accurately predict 16 out of the 216 protein expression levels present in the RPPA arrays including a phosphoprotein HER2-pY1248 (test set AUC > 0.75) (Fig. 4a, Supplementary Data 5). Clinically, in breast cancer protein expression of the estrogen receptor (ER), progesterone receptor (PR) and human epidermal growth factor receptor (HER2) direct breast cancer treatments and are routinely assessed by immunohistochemical (IHC) staining. These three proteins expression assessed by either RPPA and/or IHC were all highly predictable by our models (AUC > 0.75) (Fig. 4a–d). Among the other 13 highly predictable proteins, many of them were related to cell cycle including CCNB1, CCNE1, and FOXM1. Another interesting predictable protein was ASNS (test set AUC = 0.82); ASNS has recently been shown to play an important role in breast cancer metastasis, where its high expression was linked to an increased metastatic potential for lung metastases, and which represents a possible therapeutic target^32,33^.

In breast cancer, the most critical therapeutic biomarkers are ER, PR, and HER2 scored for by immunohistochemistry. For HER2 prediction, *ERBB2* and 17q were selected with the largest coefficients, by both the model for HER2 RPPA protein expression, and by the model guided by HER2 clinical IHC status (Supplementary Fig. 7e, f). In contrast, protein expression of ER cannot be explained by *ESR1* copy number changes since *ESR1* copy number gain/loss is rare^34^. Yet our Elastic Net models were able to accurately predict ER RPPA protein expression (AUC 0.82) and ER clinical IHC status (AUC 0.89 on METABRIC validation set) when making use of DNA copy number information only. The feature landscapes of these two models were complex with many positive and negative predictors. Notably, luminal features 16p gain and 16q loss were included in the model, consistent with the fact that ER positivity is prevalent in luminal tumors (Fig. 4e). PR positivity is highly concordant with ER positivity, where we note many regions predicting ER expression also predicted PR expression (Supplementary Fig. 7a–d).

Finally, an increasingly common clinical assay used for cancer patients is a DNA-based gene panel assay where typically tens to hundreds of genes are DNA sequenced using massively parallel sequencing, thus giving somatic mutation status and DNA copy number values for each gene^35^. One of the most widely utilized gene panels is Foundation One, which at the time of the writing of this manuscript contained 313 genes. Using only the DNA copy number information for these 313 genes, we repeated all Elastic Net prediction models, and achieved essentially identical results and AUC values (Supplementary Fig. 8, Supplementary Data 6); thus, these complex expression and protein phenotypes can be accurately predicted when using only a small subset of the human genome.

### CNA-based predictions for somatic mutations

We next examined the ability to predict individual somatic mutations. We utilized mutation data from TCGA breast tumors that have highly confident mutation calls^36^, and we limited the analyses to the significantly mutated gene list identified by previous work as well as frequently mutated genes (frequency >5%) excluding *HLA* and *IGH* genes. Only a few mutations passed the test set AUC threshold of 0.75, namely *TP53*, *CDH1*, *MAP3K1* (Fig. 4f, g, Supplementary Data 7); *GATA3* and *PIK3CA* also had relatively high AUC values though slightly below 0.75. Both *TP53* and *CDH1* models selected CNA segments encompassing these two genes as negative predictors, consistent with their known tumor suppressor phenotypes. Luminal subtype-specific mutation models, namely *GATA3* and *MAP3K1*, selected luminal copy number changes including 16p gain. Interestingly, tumor mutation burden, defined here as the total number of mutations per sample that has been shown to be related to immune therapy response^37,38^, was highly predictable from DNA CNAs (Fig. 4h). Multiple genes involved in DNA damage repair including *MGMT* and *RAD51* were selected by the model, indicating their loss correlated with increased tumor mutation burden (Fig. 4i)

### Subtype-specific predictions for gene signatures

**Fig. 5:**
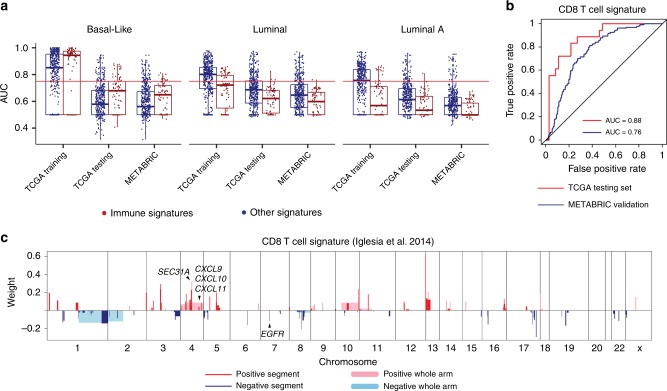
Intrinsic subtype-specific CNA-based Elastic Net models in breast cancer. **a** Box and whisker plots indicating the median score (horizontal line), the interquartile range (IQR, box boundaries) and 1.5 times the IQR (whiskers) of AUC values for predicting gene signatures within Basal-like (n = 185), Luminal (*n* = 853) and Luminal A (*n* = 556) samples; note that the plots are stratified into immune signatures (*n* = 78) and other signatures (*n* = 465). The red horizontal line indicates AUC = 0.75. **b** ROC curves and corresponding AUC values of TCGA test set and METABRIC validation set for predicting CD8 T cell expression signature within Basal-like samples. **c** Selected CNA segments and/or whole chromosomal arms and their coefficients of prediction model for CD8 T cell expression signature within Basal-like samples.

To investigate if molecular subtype affects the predictability of gene signatures, we performed identical Elastic Net analyses as described above, but only applied to Basal-like subtype tumors, Luminal A subtype tumors, or Luminal tumors (HER2-Enriched, Luminal A, and Luminal B combined). Prediction accuracies differed by subtype in many cases (Supplementary Data 8). One striking example was that some immune cell signatures were uniquely predictable within Basal-like tumors only (Fig. 5a). Specifically, a CD8 T-cell signature^39^ had AUC values of 0.74 and 0.88 when using all samples versus Basal-like samples (Fig. 5b). The segments selected to predict this signature encompassed genes encoding CD8 T-cell chemokines *CXCL9*, *CXCL10*, *CXCL11* and a gene that relates to chemokine secretion *SEC31A*, both of which affect T-cell trafficking^40^. Interestingly, *EGFR* was selected as a negative predictor, providing evidence that tumor intrinsic mechanisms shape tumor immune microenvironment^41^ (Fig. 5c). This finding demonstrates the heterogeneity underlying different subtypes and provides insights on prioritizing Basal-like tumors for immunotherapy.

### Predictions for gene signatures in lung cancer

To evaluate the generalizability of our Elastic Net modeling strategy, we evaluated prediction models using TCGA lung cancer data including both lung adenocarcinoma (LUAD) and lung squamous cell carcinoma (LUSC)^42,43^, again using gene expression signatures. We first applied the DNA-based prediction models derived from breast cancers onto lung cancers. Results identified 37 signatures that passed the AUC threshold of 0.75 across the lung training set, lung test set and breast cancer test set (Supplementary Fig. 9a, Supplementary Data 9). Not surprisingly, most of these signatures were amplicon signatures, however, two were related to *TP53* mutation status and PTEN/PI3K pathway activity. CNA segments and/or whole chromosomal arms selected by models built on breast cancer or lung cancer for a *TP53* status signature were similar (Supplementary Fig. 9d, e), indicating that the Elastic Net approach was able to consistently select the most relevant features. Lastly, a larger number of signatures were found to be predictable if trained on lung cancer data, suggesting some models may be tumor type dependent, while others may be tumor type independent (i.e., *TP53*). We also evaluated if DNA CNA-based Elastic Net model can successfully classify the two lung cancer histologies. Results showed that we can classify LUAD vs. LUSC with very high accuracy (AUC = 0.98 and 0.97 for training and test set), consistent with previous finding that LUAD and LUSC have distinct somatic drivers^44^ (Supplementary Fig. 9c, f).

### Pan-Cancer predictions for gene signatures

**Fig. 6:**
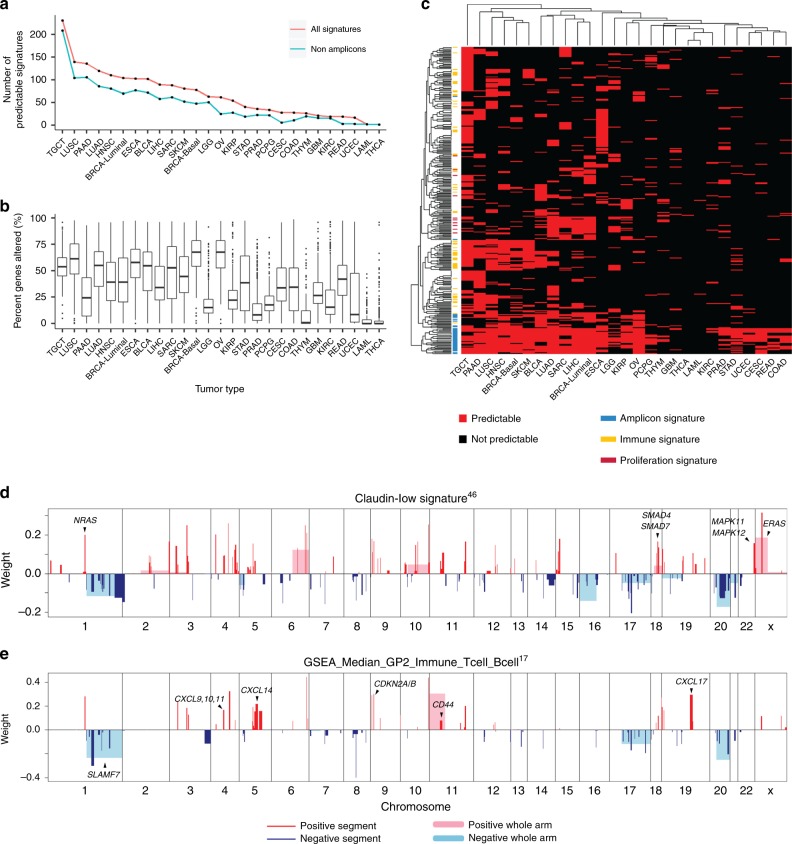
Pan-cancer DNA copy number alteration-based Elastic Net models for gene signatures. **a** Line plots indicate the number of highly predictable signatures (i.e., AUC > 0.75) (red) and highly predictable non-amplicon signatures (green) in each tumor type. **b** Box and whisker plots indicating the median score (horizontal line), the interquartile range (IQR, box boundaries) and 1.5 times the IQR (whiskers) of the percentage of copy number altered genes in each tumor type. **c** Heatmap shows the predictability of each gene signature in each tumor type. Red indicates predictable and black indicates not predictable. Tumors and gene signatures are clustered by hierarchical clustering using Euclidean distance and complete linkage. **d**, **e** Selected CNA segments and/or whole chromosomal arms and their coefficients of the multi-tumor prediction model for Claudin-low signature (**d**) and immune signature (**e**).

Further extending the approach to a total of 25 tumor types from TCGA that have multi-platform data and at least 100 samples^4^ identified successful models with high accuracy (AUC > 0.75) for multiple tumor types. Not surprisingly, there were more gene signatures that were highly predictable in tumor types that have more CNA events (Fig. 6a, b). Hierarchical clustering of tumor types based on the predictability of gene signatures revealed informative tumor subgroupings consistent with previous Pan-Cancer findings^4^ (Fig. 6c) including a Pan-Squamous group. Basal-like breast cancer clustered with squamous cancers including LUSC and Head and Neck squamous cell carcinoma (HNSC). Many immune-related signatures were uniquely predictable in these tumor types including the aforementioned CD8 T-cell signature as well as PD1 and CTLA4 signaling pathways. On the other hand, Luminal breast cancer clustered with LUAD and Bladder Urothelial Carcinoma (BLCA), where multiple signatures measuring proliferation rate including the RB-LOH signature were highly predictable. Lastly, amplicon signatures were universally predictable across tumor types that have high percentage of copy number altered genes.

Mesenchymal features in epithelial cancers are typically associated with poor outcomes and therapy resistance, and have been shown to be associated with a breast cancer subtype called Claudin-low^45^. A Claudin-low/mesenchymal signature^46^, was highly predictable within 9/25 tumor types, therefore we investigated CNA regions that are universally important in predicting this signature by building a model using the combined data from these 9 tumor types (Supplementary Data 10). The resulting model had a training set AUC of 0.8 and test set AUC of 0.74. CNA regions selected by this model highlighted many RAS/MAPK pathway components including a less-known gene *ERAS* (Fig. 6d), consistent with the finding that its forced expression induced EMT in human mammary gland cells^47^. Similarly, an immune signature^17^ was predictable across 8 tumor types with a number of T cell chemokine gene chromosomal regions selected (Fig. 6e). These results demonstrated that our Elastic Net approach was able to robustly build predictive models for key gene signatures when using multiple genomically related tumor types.

## Discussion

The ability to predict key tumor phenotypes, like mutation status or biomarker levels or complex expression phenotypes, is critical to understanding the biological complexity of solid epithelial cancers. Nowadays for breast cancer, protein expression analysis is required for ER, PR, and HER2, and small panel (i.e., 21–70 genes) gene expression tests are common. For lung cancer, DNA-based gene panel testing is included within the standard of care, and expression analyses for proteins (i.e., PDL1) are growing in prominence, in large part due to immunotherapy. Many solid epithelial cancers, particularly breast and lung, are thought to be at least partially DNA copy number driven because a large number of copy number events occur, and many are known genetic drivers^48,49^. We, therefore, reasoned that many key tumor phenotypes might be predictable when using the diversity of DNA copy number changes when examining a proposed copy number driven tumor type. To address this hypothesis, we used an extensive archive of manually curated gene expression signatures taken from multiple publications, to study tumor phenotypes and estimate their predictability. We investigated the relationship between DNA copy number alterations and each gene expression signature through two means; first was a genome-wide association method, while the second was to build Elastic Net prediction models and assess their accuracy. The association study allowed us to find genes positively or negatively correlated DNA features to expression signatures by evaluating genes one by one. These two methods cooperatively produced a big picture of linkages between CNAs and gene signatures. We consistently found known associations between CNAs and gene signatures, including gene signatures of DNA amplifications and losses, and for gene signatures of more complex phenotypes including signaling pathway activities (i.e. TP53 and EGFR), and gene signatures of cellular proliferation status; in fact, we were able to predict many of these signatures with very high accuracy (AUC > 0.9) on a true test set that even used different gene expression and DNA copy number technologies (i.e., METABRIC). Taken together each gene signature’s association landscape and Elastic Net feature landscape provides CNA regions for further investigation for potential genetic drivers. In addition, further application of our Elastic Net modeling strategy to a variety of other molecular phenotypes including molecular intrinsic subtypes, protein expression levels and somatic mutation status (including tumor mutation burden) revealed the ability to accurately predict many key phenotypes in breast cancer. These models may be clinically useful and could provide an orthogonal approach for calling key features like ER, PR, and HER2 status in breast cancer, especially in equivocal cases, given the growing use of DNA exomes and somatic mutation gene panels in the cancer clinic.

For the analyses presented here, we chose to dichotomize the expression signatures into the highest tertile versus the bottom two tertiles; however, we also evaluated Elastic Net models where the expression signatures were treated as continuous variables, and these were also successful for those models that showed high AUCs when tested as dichotomous variables (Supplementary Fig. 10), albeit with lower but still acceptable accuracies. Thus, it may even be feasible to predict quantitative traits, in addition to the simple high versus low as was done for the majority of our predictors.

Many commercial gene panel tests have been developed with the goal of improving precision medicine. Using DNA CNA information of only 313 genes that can be derived from Foundation One testing, gene expression signature prediction and protein expression prediction accuracies remained the same when compared to that using whole exome of CNA values. The 313 genes have been selected as highly cancer relevant and reported to be important in tumorigenesis. This result suggests a small part of the genome accounts for a large part of the predictive power of cancer phenotypes seen in some solid epithelial cancer types. This also sheds light on the application of Elastic Net models in the clinic. For example, various proliferation signatures, including the RB-LOH signature evaluated here, might serve as a potential biomarker for CDK4/6 inhibitors which target the RB/E2F pathway^50^. Our Elastic Net model for RB-LOH signature could be used to stratify patients into those with high proliferation rates, which typically identifies those with RB loss, and for whom then a CDK4/6 inhibitor would not be recommended. Further validation is needed to confirm this specific hypothetical application, however, if validated, then a whole new set of prognostic and predictive biomarkers could be read out from existing DNA-based gene panels, thus providing more guidance for precision medicine at no additional cost.

Lastly, we showed the generalizability of our approach through a Pan-Cancer analysis of 23 different tumor types. Consistent with our hypothesis, a variety of gene signatures besides amplicon signatures were predictable in tumor types that have many copy number changes. Tumor types that have been shown to share similar features had similar patterns of signature predictability. More importantly, those shared key features were often highly predictable such as immune features in squamous/basal-like tumors and proliferation rate in adenocarcinomas (i.e., lung and breast luminal).

Collectively our results demonstrate the ability to build CNA-based predictors for multiple key cancer phenotypes for breast and non-small cell lung cancer patients. While most research focuses on finding genetic drivers of tumorigenesis, our work carries important implications that critical complex tumor phenotypes can be predicted using DNA information, which could be potentially used in the clinic.

## Methods

### Gene expression data

Illumina HiSeq 2000 RNA sequencing data for human breast cancer and lung cancer (both Lung Adenocarcinoma and Lung Squamous Cell Carcinoma) were acquired from The Broad Institute TCGA GDAC Firehose^4^. Illumina HT-29 v3 expression data for the METABRIC project (*n* = 1992 samples) were acquired from the European Genome-phenome Archive at the European Bioinformatics Institute^3^. For TCGA breast cancer and lung cancer gene expression data, gene-level RNA-Seq reads were upper-quartile normalized and log2 transformed, filtered to genes that were expressed in over 70% of samples, median centered and sample-wise standardized within each data set. For METABRIC microarray gene expression data, acquired data were filtered to genes that were expressed in over 70% of samples and were median centered for each gene and standardized for each sample. Gene expression data for all other tumor types were downloaded from GDC PanCanAtlas publication site (https://gdc.cancer.gov/about-data/publications/pancanatlas). For each tumor type, gene expression data were filtered to genes that were expressed in over 70% of samples, median centered and sample-wise standardized within each tumor type.

To determine breast cancer intrinsic PAM50 subtypes for TCGA breast cancer data, the TCGA RNA-seq data were first subsampled to match the ER distribution of the training set used for PAM50. Then the entire data set were adjusted to the median gene expression calculated for the PAM50 genes determined from the ER balanced subset. Intrinsic subtyping was then done as previously described^36^. For METABRIC, median-centered and standardized gene expression were used to calculate correlation to each subtype centroid^11^ (Supplementary Data 11).

### DNA copy number data

GISTIC2 gene-level copy number data for human breast cancer and lung cancer were acquired from The Broad Institute TCGA GDAC Firehose with no further processing (all_data_by_genes.txt). For the METABRIC project, copy number segmentation data using circular binary segmentation (CBS) algorithm were acquired from the European Genome-phenome Archive^3^. Using Ensembl 54 (hg18) genome build, gene-level copy number score were derived through the extreme method as used in GISTIC2 (ref. ^51^): Genes that fell completely within a CBS-identified copy number segment were assigned corresponding segment value. Genes that overlapped with multiple segments were assigned the greatest amplification or the least deletion value among the overlapped segments. Genes with no overlapping segments were excluded from further analyses. GISTIC2 gene-level copy number data for all other tumor types were downloaded from GDC PanCanAtlas publication site with no further processing (https://gdc.cancer.gov/about-data/publications/pancanatlas).

### Protein expression data

Normalized protein expression data for human breast cancer were acquired from The Broad Institute TCGA GDAC Firehose with no further processing.

### Mutation data

Mutation Annotation Format (MAF) data from 2015 TCGA Lobular Breast Cancer dataset were used^36^. MAF file was first filtered to only include the following variant classifications: Frame_Shift_Del, Frame_Shift_Ins, In_Frame_Del, In_Frame_Ins, Missense_Mutation, Nonsense_Mutation, Nonstop_Mutation, RNA,Splice_Site, Translation_Start_Site. A binary gene by sample matrix of 1 indicating any mutation and 0 indicating no mutation was then constructed based on the filtered MAF. Mutation load for each sample was then determined by the total number of mutated genes in that sample.

### Gene expression signatures

A panel of 543 previously published gene expression signatures were used to fully characterize cancer phenotypes. These 543 signatures were obtained from multiple publications or GSEA^52^ and were partially summarized by Tanioka et al.^13^. The complete list of genes in each signature and their references is shown in Supplementary Data 1. Signature scores were calculated in a manner consistent with their derivation. For 504 signatures with homogeneous expression across the genes, median expression value was used as signature score. The rest of the signatures were based on correlation to predetermined gene centroids or based on published algorithms. For correlation-based signatures, all predetermined training sets are available to download through our GitHub repository (See code availability). For each such signature, DWD^53^ was used to first merge gene expression matrix with corresponding training set and then Pearson/Spearman correlation/Euclidean distance was computed for each sample in the merged data. For several algorithm-based signatures, corresponding R code is provided to calculate each signature (See code availability). All 543 signatures were applied to TCGA breast cancer and lung cancer data as well as METABRIC data. 504 median-expression based signatures were applied to Pan Cancer data.

### Identification of gene signature-specific CNAs

To identify associations between CNAs and gene expression signatures, two independent statistical tests were used^8^ on TCGA breast cancer cohort with matched gene expression and copy number data excluding all Normal-like samples (*n* = 1038). For each signature, a spearman rank correlation, both positive and negative, was used to compare gene-level copy number score with signature score. A one-sided Fisher’s exact test was used to compare either frequency of CNA gain or loss in samples with high signature score (top quartile) and those with low signature score (bottom three quartiles). For each analysis, Benjamini–Hochberg multiple testing correction was used to adjust *p*-values for each signature across all genes. Significant threshold was set to 0.01 to identify genes that were significant in both analyses.

To identify subtype-adjusted associations between CNAs and each gene expression signature, a linear model was used instead of the spearman rank correlation to take into account molecular subtype as confounding variables: signature ~ CNA + (1|Basal) + (1|HER2) + (1|LumA) + (1|LumB). Positive/negative correlation was determined by the coefficient of CNA and *p*-value in the model. Fisher’s exact test, Benjamini–Hochberg multiple testing correction and significant threshold of 0.01 were done the same way as described above.

### Building Elastic Net prediction models

We used Elastic Net modeling approach, which is a regularized regression method that linearly combines the L1 and L2 penalties of the Ridge Regression and Least Absolute Shrinkage and Selection Operator (LASSO), to build DNA CNA-based predictors of cancer phenotypes^9^. Generally, gene-level CNA scores were first collapsed to segment-level CNA scores. The complete list of genes in each segment is shown in Supplementary Data 2^18–22^. Each segment score was calculated as the mean CNA score across genes within the segment. For each cancer phenotype, total sample was split into 70% training set and 30% testing set (R package sampling) stratified by clinical variables: overall survival, gender, race, ER status, PR status and HER2 status, histological subtype, pathologic stages and molecular subtype when available. Models were built on training set only. Tuning grid were determined with alphas over a range from 0.1 to 1 by 0.1 and a sequence of 100 lambdas. The minimum and maximum of lambda values were determined by fitting generalized linear models with each alpha value on training set (R package glmnet). 200 rounds of Monte-Carlo cross validation with default training percentage of 0.75 (R package caret) were used to select the tuning parameters. The optimal parameter combination was determined to have the best classification accuracy. Model with the optimal parameters was then applied to test set and other validation sets if available. Receiving operating characteristics (ROC) curves were constructed and area under ROC curve (AUC) values were used to evaluate model performances (R package ROCR). We consider phenotypes with AUC values above 0.75 as highly predictable.

For predicting gene expression signatures, protein expression, and mutation load that had continuous scores, models were built to classify samples with high scores (top third) versus low scores (bottom two-thirds). For molecular subtype, clinical receptor status, cancer histology, and mutations that had binary outcomes, models were built to classify each outcome. For breast cancer gene expression signatures, Normal-like samples were excluded (*n* = 1038) as in association tests described above. For somatic mutations, all *IGH* and *HLA* genes were removed and only genes that have mutation frequency greater than 5% and/or significantly mutated genes identified in 2015 TCGA Lobular Breast Cancer analysis^36^ were included.

For subtype-specific gene signature predictions, the same Elastic Net model approach was repeated within samples of a particular subtype, split into 70% training and 30% testing, and models were applied to METABRIC samples with the same subtype.

For gene signature and histology prediction using the non-small cell lung cancer data, the whole TCGA lung data set was used that combined both LUAD (*n* = 498) and LUSC (*n* = 512), which were split into training and testing sets balanced for clinical variables: overall survival, gender, pathological stages and histology (LUAD or LUSC). Models were built on training set and applied to testing set. Models built on TCGA breast cancer training set were also applied to the whole lung data set. Models were also built within LUAD and LUSC separately.

For Pan Cancer gene expression signature predictions, we limited tumor types with at least 100 samples that had RNA, DNA, and clinical data. 504 median expression-based gene signatures were applied to each tumor type. For each signature prediction in each tumor type, total sample was split into 70% training set and 30% testing set, balanced for gender, race and overall survival. Models were then built on training set and applied to testing set to get training and testing AUC values. For the multi-tumor prediction of Claudin-low and immune signature, DNA data and binarized signature level data were first combined across tumor types that each signature was highly predictable and then split into 70% training and 30% testing balanced for gender, race, overall survival, and tumor type. Models were built on training set and applied to test set.

We used permutations to assess the use of 0.75 as the threshold to define ‘highly predictable’. Tumor labels were permuted 100 times for 16 representative phenotypes, namely RB-LOH signature, Basal signaling signature, estrogen signaling signature, HER1-C2 signature, ER/PR/HER2 IHC status, *TP53* mutation, tumor mutation load and intrinsic molecular subtypes. The same Elastic Net modeling process for each phenotype were conducted as described above. Briefly for each phenotype, during each permutation, all samples were split into 70% training and 30% test sets stratified by clinical variables. Models were built using training set and test set AUCs were used to compare with the threshold of 0.75.

To look at the features selected by each prediction model, coefficients of CNA segments were re-mapped to genes within each segment and plotted. Summary of all Elastic Net models including coefficients of CNA segments and AUC values are reported in Supplementary Data 3–10.

### Survival analysis in METABRIC

To investigate the prognostic value of CNA-based Elastic Net model of some known prognostic signatures, we applied research-based versions coming from METABRIC microarray data, for three clinically used assays and four other representative signatures and their prediction models, namely OncotypeDX recurrence score, MAMMAPRINT 70-GENE recurrence score, Prosigna risk of recurrence score, RB-LOH signature, basal-signaling signature, estrogen-signaling signature and HER1-C2 signature to 1689 METABRIC samples excluding all Normal-like samples. 10-year breast cancer-specific survival data were derived by censoring patients that had death unrelated to breast cancer or that had survival time over 10 years. For each signature, patients were divided into high/low groups according to observed signature score (top third vs. bottom two-thirds) as well as predicted probability by Elastic Net prediction model (top third vs. bottom two-thirds). Kaplan–Meier curves were drawn (R package survival) and log rank *p*-values were calculated.

### Reporting summary

Further information on research design is available in the Nature Research Reporting Summary linked to this article.

## Supplementary information

Description of Additional Supplementary Files

Supplementary Data 1

Supplementary Data 2

Supplementary Data 3

Supplementary Data 4

Supplementary Data 5

Supplementary Data 6

Supplementary Data 7

Supplementary Data 8

Supplementary Data 9

Supplementary Data 10

Supplementary Data 11

Supplementary Data 12

Supplementary Information

Reporting Summary

## Data Availability

The TCGA data referenced during the study are available from the TCGA website (the Broad Institute TCGA GDAC Firehose: https://gdac.broadinstitute.org/). The METABRIC data is available from the European Genome-phenome Archive at the European Bioinformatics Institute (https://www.ebi.ac.uk/ega/). All the other data sets supporting the findings of this study are available within the article, the Supplementary information tables and our GitHub repository and from the corresponding author upon reasonable request.

## Code availability

All code supporting the current study is deposited in GitHub (https://github.com/xyouli/DNA-based-predictors-of-non-genetic-cancer-phenotypes). All computational analyses are done using public R packages.
