# Supplementary material for: Genetic determinants of the molecular portraits of epithelial cancers
Source: Nat Commun. 2019 Dec 11;10:5666. doi: 10.1038/s41467-019-13588-2 (PMC6906458; doi:10.1038/s41467-019-13588-2)
Supplement: Supplementary file 1 — Description of Additional Supplementary Files [file 41467_2019_13588_MOESM1_ESM.pdf]

Description of Additional Supplementary Files

File Name: Supplementary Data 1
Description: Annotation of Gene Expression Signatures.

File Name: Supplementary Data 2
Description: Annotation of copy number segments.

File Name: Supplementary Data 3
Description: Summary of Elastic Net models for gene expression signatures.

File Name: Supplementary Data 4
Description: Summary of Elastic Net models for molecular subtypes and histology in breast cancers.

File Name: Supplementary Data 5
Description: Summary of Elastic Net models for protein expressions and clinical receptor statuses in breast cancers.

File Name: Supplementary Data 6
Description: Summary of AUC values for the prediction of gene expression signatures and protein expressions using Foundation One genomic test genes in TCGA breast cancer.

File Name: Supplementary Data 7
Description: Summary of Elastic Net models for somatic mutations in breast cancers.

File Name: Supplementary Data 8
Description: Summary of subtype-specific signature predictions in breast cancers.

File Name: Supplementary Data 9
Description: Summary of Elastic Net models for gene expression signatures in lung cancers.

File Name: Supplementary Data 10
Description: Summary of Pan Cancer signature predictions.

File Name: Supplementary Data 11:
Description: PAM50 subtypes for METABRIC.

File Name: Supplementary Data 12
Description: List of amplicon signatures.
