# Supplementary material for: Genetic determinants of the molecular portraits of epithelial cancers
Source: Nat Commun. 2019 Dec 11;10:5666. doi: 10.1038/s41467-019-13588-2 (PMC6906458; doi:10.1038/s41467-019-13588-2)
Supplement: Supplementary file 14 — Supplementary Information [file 41467_2019_13588_MOESM14_ESM.pdf]

Genetic Determinants of the Molecular Portraits of Epithelial Cancers

Xia et al.

Supplementary Information

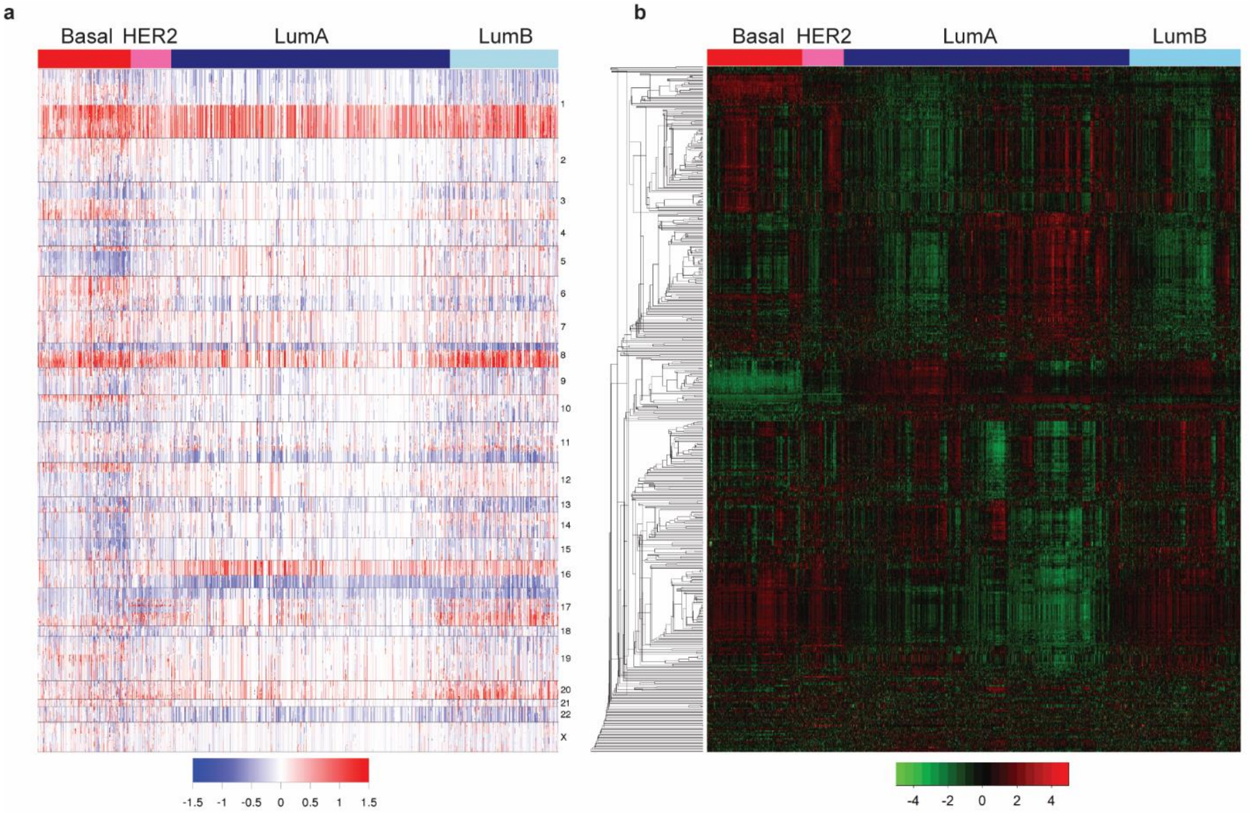

Supplementary Fig. 1 Patterns of DNA CNAs and gene expression signatures in breast cancer. a, Heatmap showing DNA CNAs with red indicating gain and blue indicating loss. Samples are ordered on the X axis according to molecular subtype. Genes are ordered on the Y axis according to chromosomal location. **b**, Heatmap showing gene expression signatures. Samples are ordered on the X axis according to molecular subtype. Gene signature scores are median centered and clustered by centroid linkage hierarchical clustering based on Pearson correlation.

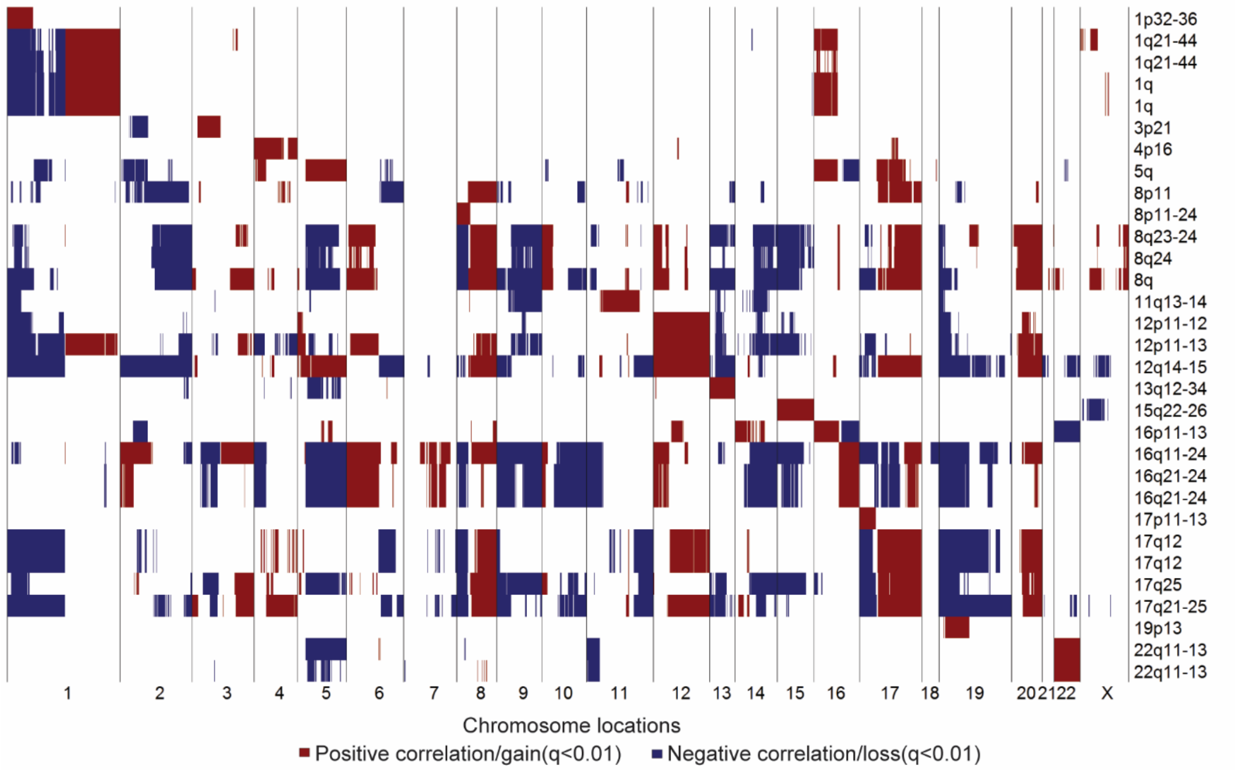

Supplementary Fig. 2 Patterns of associations between DNA CNAs and amplicon signatures. Genes that had a positive correlation and increased frequency of copy number gains ($q < 0.01$) are shown in red and those that had a negative correlation and an increased frequency of copy number losses ($q < 0.01$) in samples with high signature scores (top quartile) are shown in blue. Each amplicon signature has positive associations with its corresponding amplicon.

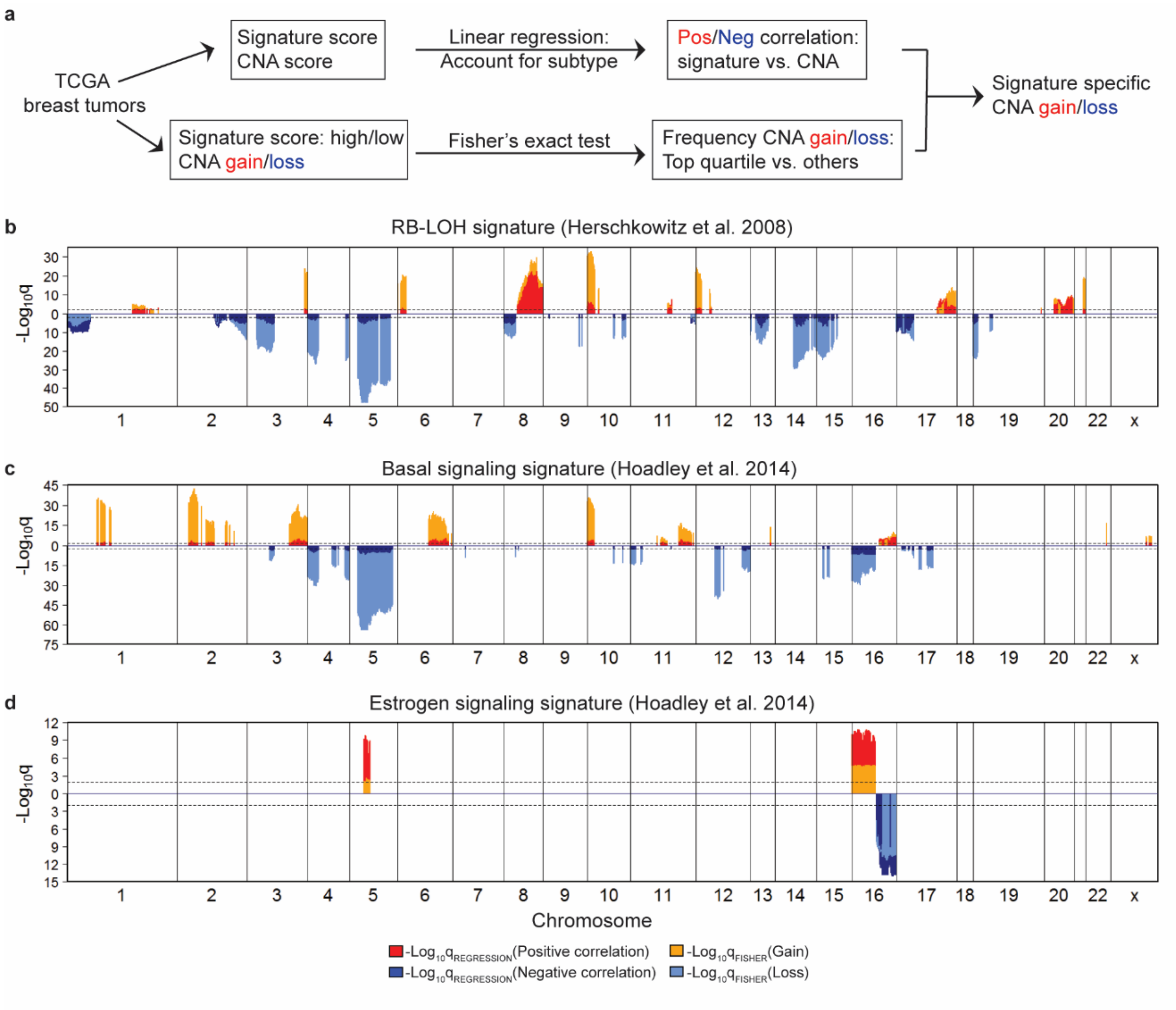

Supplementary Fig. 3 Identification of subtype-adjusted gene signature-specific CNAs in breast cancer. a, Schematic overview of the strategy used to identify CNAs associated with gene signatures accounting for molecular subtypes. Gain/loss indicates DNA copy number gains or losses; Pos/Neg indicates positive or negative association. **b-d**, Linear regression accounting for molecular subtype was used to identify genes positively (red) or negatively (dark blue) associated with gene signatures, and Fisher's exact test was used to compare the frequency of copy number gains (orange) or losses (light blue) for RB-LOH (**b**), Basal signaling (**c**), and Estrogen signaling (**d**) Gene Program signatures. Dashed lines indicate significance threshold (q = 0.01). Only q values for genes significant in both analyses were plotted.

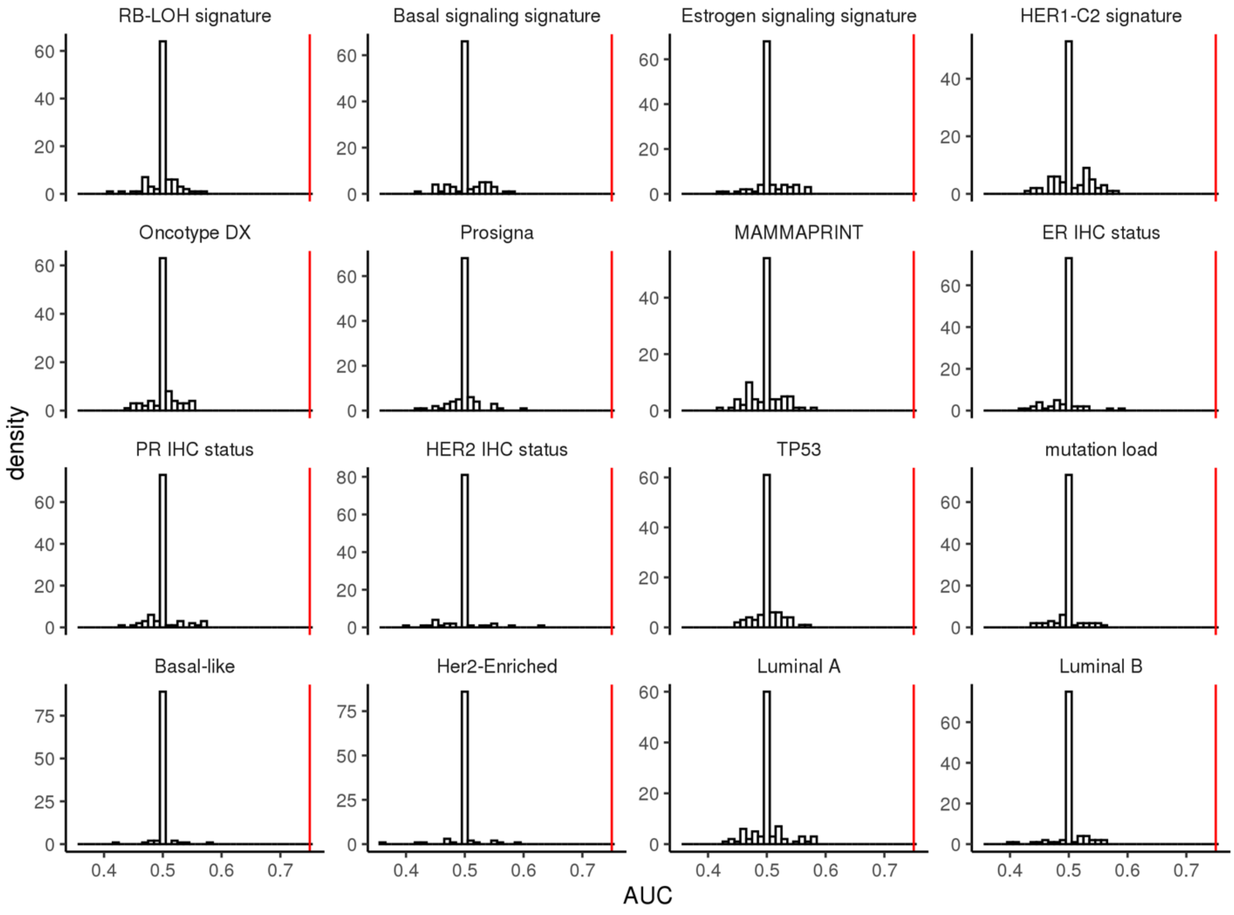

Supplementary Fig. 4 Histogram of permuted test set AUC values. Test set AUC values from 100 permutations per each phenotype, were plotted for each highly predictable gene expression signature, clinical receptor status, somatic mutation and intrinsic molecular subtypes. Red vertical line indicates AUC = 0.75, which is used as the threshold to define ‘highly predictable’.

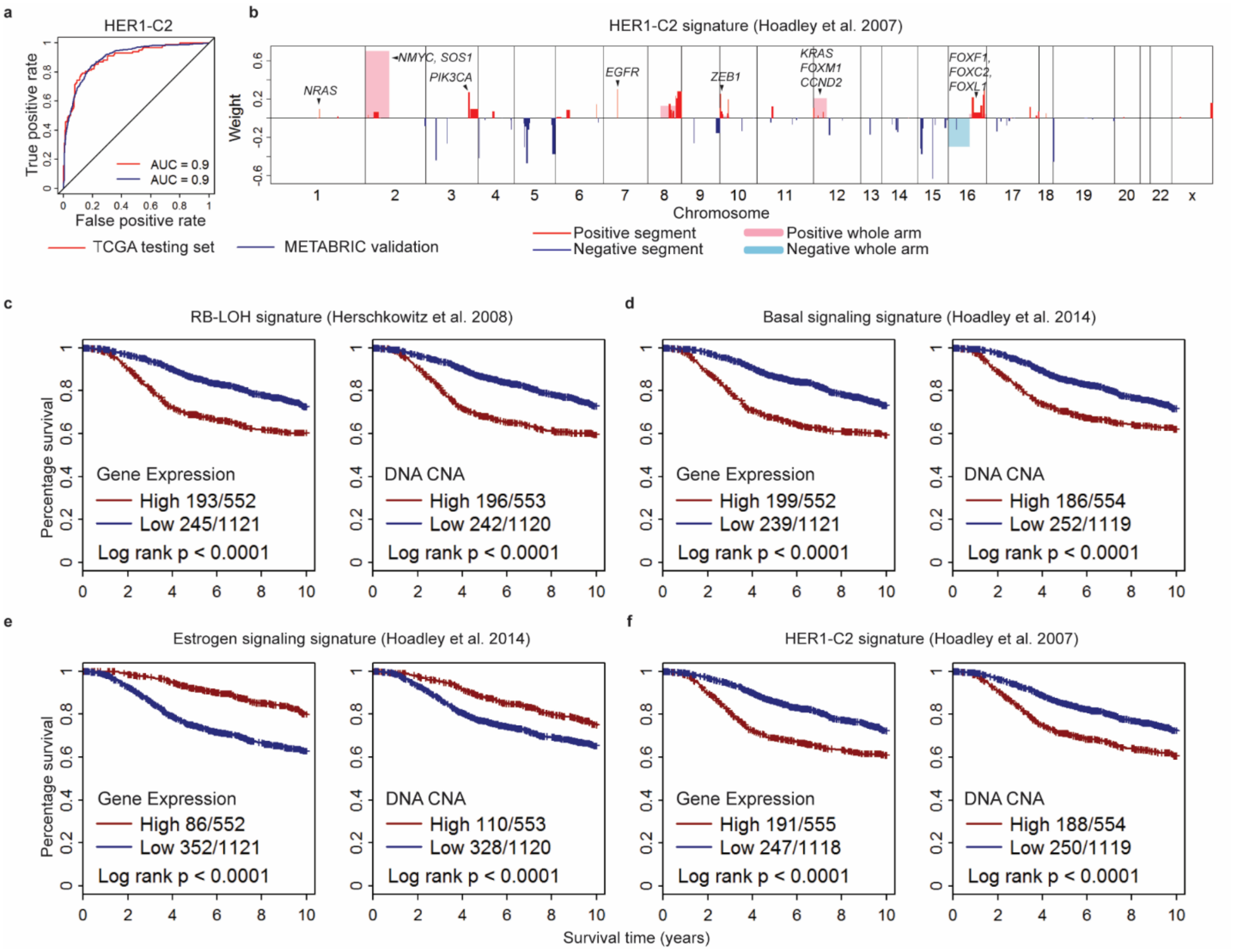

Supplementary Fig. 5 CNA-based Elastic Net prediction models for multiple key expression signatures and prognosis. a, ROC curves and corresponding AUC values of TCGA test set and METABRIC validation set for HER1-C2 signature. **b**, Selected CNA segments and/or whole chromosomal arms and their coefficients of prediction model for HER1-C2 signature. Known drivers of EGFR pathway are highlighted with black arrows. **c-f**, Kaplan-Meier curves of 10-year breast cancer-specific survival stratified by gene signature score (Gene Expression) and corresponding Elastic Net prediction model (DNA CNA) for RB-LOH (**c**), Basal signaling (**d**), Estrogen signaling (**e**) and HER1-C2 (**f**) signatures. Event statistics were indicated as number of events/total patients in both High and Low groups.

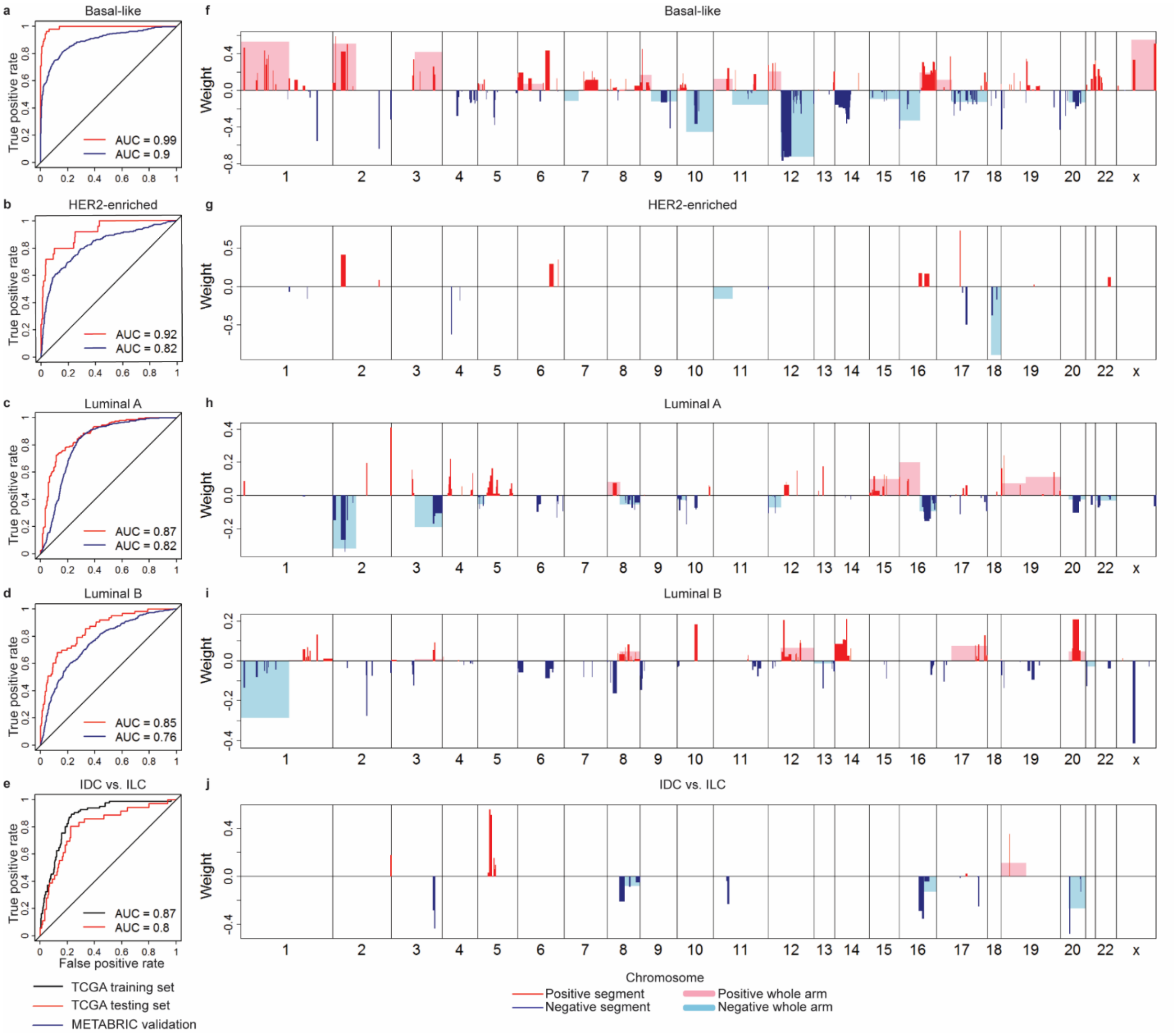

Supplementary Fig. 6 CNA-based Elastic Net prediction models for intrinsic and histological subtypes in breast cancer. a-e, ROC curves and corresponding AUC values for predicting Basal-like (**a**), HER2-enriched (**b**), Luminal A (**c**), and Luminal B (**d**) subtypes, and breast cancer histology IDC vs. ILC (**e**). **f-j,** Selected CNA segments and/or whole chromosomal arms and their coefficients of prediction model for Basal-like (**f**), HER2-enriched (**g**), Luminal A (**h**) and Luminal B (**i**) subtypes, and histology (**j**). Positive weights favor ILC classification.

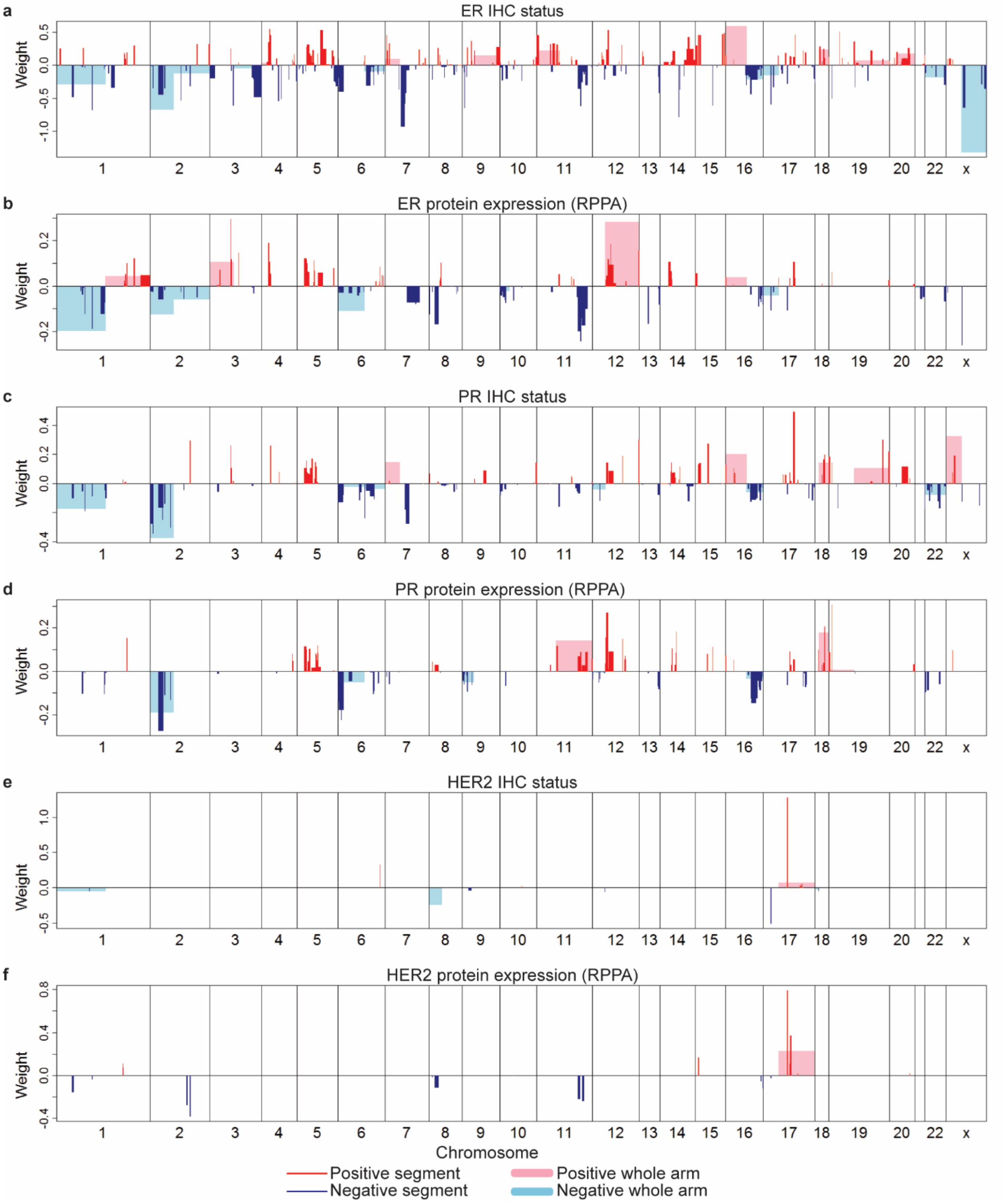

a
ER IHC status
b
ER protein expression (RPPA)
c
PR IHC status
d
PR protein expression (RPPA)
e
HER2 IHC status
f
HER2 protein expression (RPPA)
Weight
1 2 3 4 5 6 7 8 9 10 11 12 13 14 15 16 17 18 19 20 22 x
Chromosome
Positive segment
Negative segment
Positive whole arm
Negative whole arm

Supplementary Fig. 7 Selected CNA landscapes of DNA-based Elastic Net prediction models for clinical receptor status and corresponding protein expressions measured by RPPA. Selected CNA segments and/or whole chromosomal arms and their coefficients of prediction model for ER IHC status (**a**), ER RPPA expression (**b**), PR IHC status (**c**), PR RPPA expression (**d**), HER2 IHC status (**e**) and HER2 RPPA expression (**f**). Models predicting the RPPA expression and IHC status for the same protein have similar landscapes.

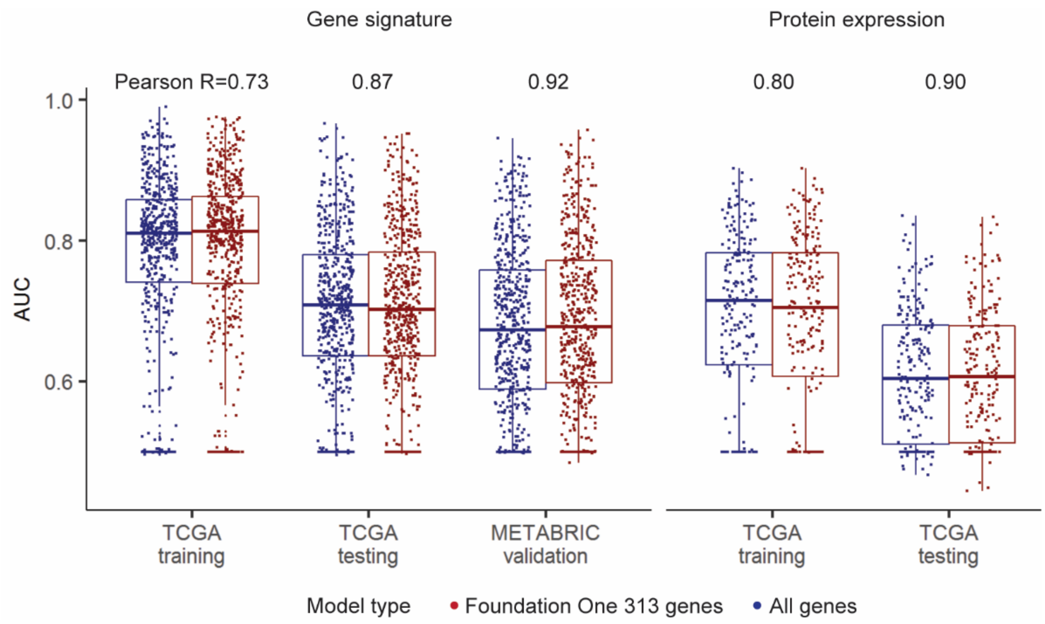

Supplementary Fig. 8 Comparison of Elastic Net model performances using predictors of all genes and Foundation One 313 gene set. Box and whisker plots indicating the median score (horizontal line), the interquartile range (IQR, box boundaries) and 1.5 times the IQR (whiskers) of AUC values for predicting gene signatures and individual protein expressions using all genes (blue) and Foundation One test 313 genes (red) in breast cancer. AUC values are highly correlated between the two categories.

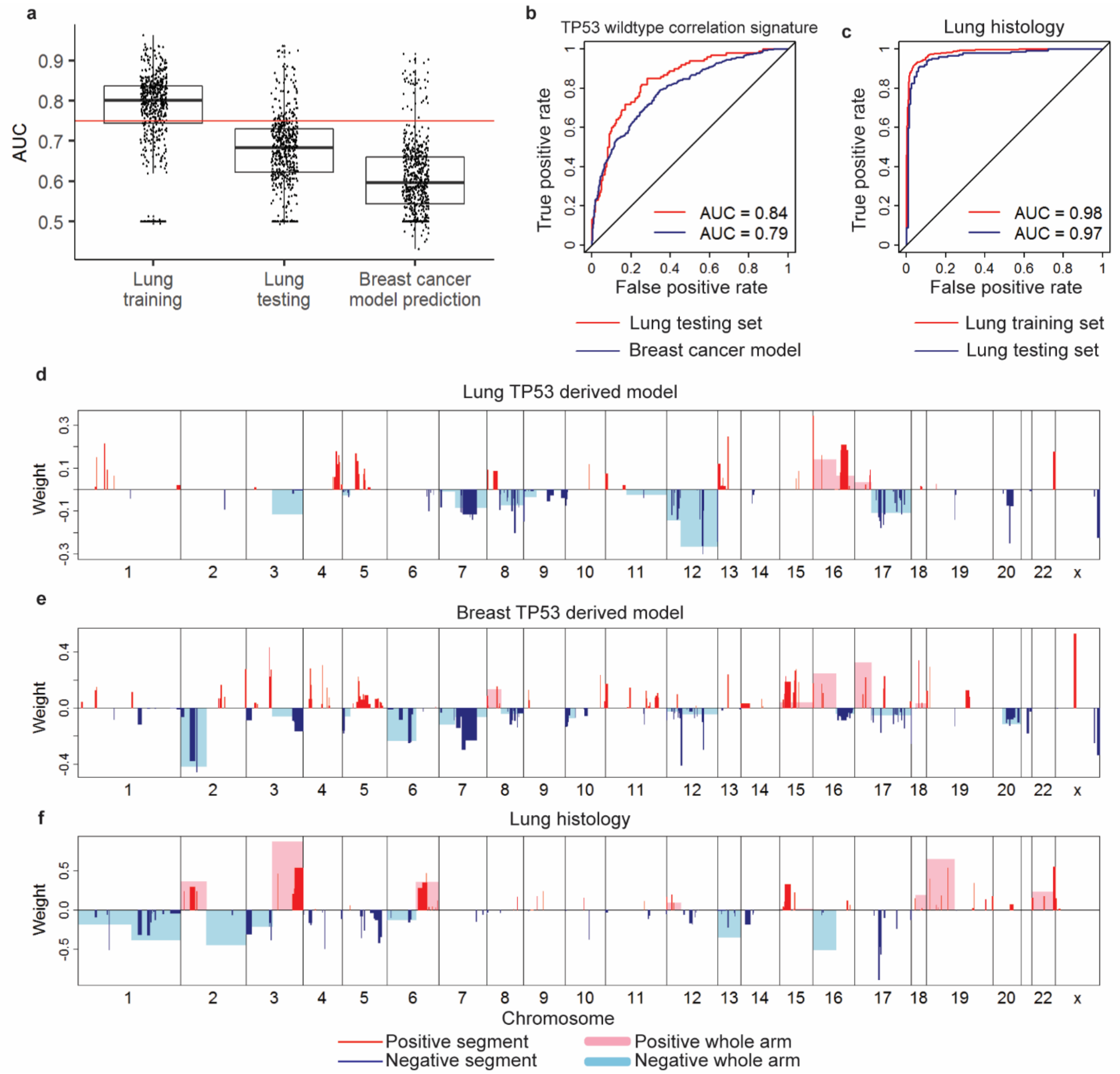
a
AUC
0.9
0.8
0.7
0.6
0.5
Lung training
Lung testing
Breast cancer model prediction
b
TP53 wildtype correlation signature
True positive rate
False positive rate
AUC = 0.84
AUC = 0.79
Lung testing set
Breast cancer model
c
Lung histology
True positive rate
False positive rate
AUC = 0.98
AUC = 0.97
Lung training set
Lung testing set
d
Lung TP53 derived model
Weight
e
Breast TP53 derived model
Weight
f
Lung histology
Weight
Chromosome
Positive segment
Negative segment
Positive whole arm
Negative whole arm

Supplementary Fig. 9 CNA-based Elastic Net prediction models for gene signatures in lung cancer. **a**, Box and whisker plots indicate AUC values for predicting gene signatures in lung cancers using models built on lung cancer data (Lung training and Lung testing on X axis) and that built on breast cancer data (Breast cancer model prediction on X axis). Red horizontal line indicates AUC = 0.75. **b**, ROC curves and corresponding AUC values for predicting a *TP53* wild type status signature showing that both models built on lung cancer data and breast cancer are successful (AUC > 0.75). **c**, ROC curves and corresponding AUC values for predicting lung histology, LUAD vs. LUSC. **d-e**, Selected CNA segments and/or whole chromosomal arms and their coefficients of prediction models built on lung cancer (**d**), and breast cancer (**e**), for a *TP53* status signature show similar feature landscapes. **f**, Selected CNA segments and/or whole chromosomal arms and their coefficients of prediction model for classifying lung histology, LUAD vs. LUSC. Positive weights favor LUSC classification.

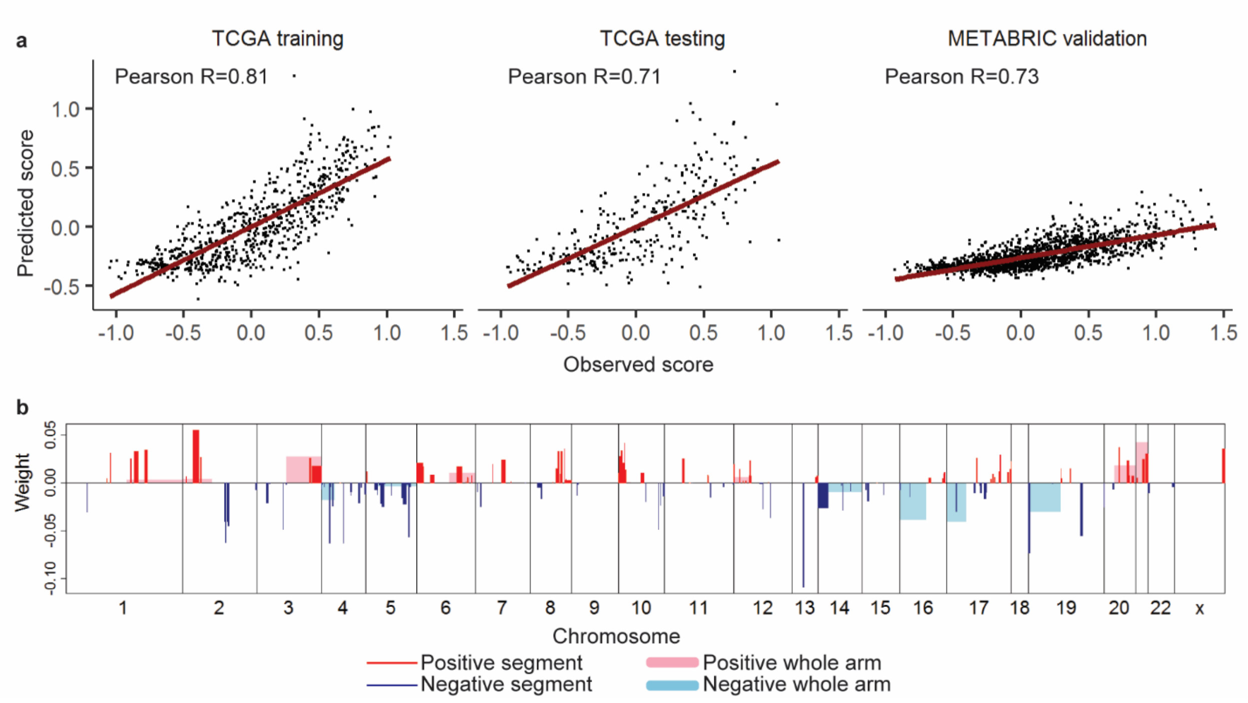

Supplementary Fig. 10 CNA-based Elastic Net prediction for continuous RB-LOH signature score in breast cancer. a, Scatter plot of predicted RB-LOH signature score against observed signature score in TCGA training set, TCGA test set and METABRIC validation set. Red line is fitted regression line. Pearson correlations are indicated. **b**, Selected CNA segments and/or whole chromosomal arms and their coefficients of the prediction model.
